# Quantifying Fatigue in the Rugby Codes: The Interplay Between Collision Characteristics and Neuromuscular Performance, Biochemical Measures, and Self-Reported Assessments of Fatigue

**DOI:** 10.3389/fphys.2021.711634

**Published:** 2021-10-29

**Authors:** Mitchell Naughton, Scott McLean, Tannath J. Scott, Dan Weaving, Colin Solomon

**Affiliations:** ^1^School of Health and Behavioural Sciences, University of the Sunshine Coast, Sippy Downs, QLD, Australia; ^2^Centre for Human Factors and Sociotechnical Systems, University of the Sunshine Coast, Sippy Downs, QLD, Australia; ^3^New South Wales Rugby League, Sydney Olympic Park, NSW, Australia; ^4^Carnegie Applied Rugby Research Centre, Leeds Beckett University, Leeds, United Kingdom; ^5^Leeds Rhinos Rugby League Club, Leeds, United Kingdom

**Keywords:** rugby, fatigue, recovery, muscle damage, external load, neuromuscular, biomarker, subjective

## Abstract

Locomotor and collision actions that rugby players complete during match-play often lead to substantial fatigue, and in turn, delays in recovery. The methods used to quantify post-match fatigue and recovery can be categorised as subjective and objective, with match-related collision characteristics thought to have a primary role in modulating these recovery measures. The aim of this review was to (1) evaluate how post-match recovery has been quantified in the rugby football codes (i.e., rugby league, rugby union, and rugby sevens), (2) to explore the time-course of commonly used measures of fatigue post-match, and (3) to investigate the relationships between game-related collisions and fatigue metrics. The available evidence suggests that upper-, and lower-body neuromuscular performance are negatively affected, and biomarkers of muscular damage and inflammation increase in the hours and days following match-play, with the largest differences being at 12–36 h post-match. The magnitude of such responses varies within and between neuromuscular performance (Δ ≤ 36%, *n* = 13 studies) and tissue biomarker (Δ ≤ 585%, *n* = 18 studies) measures, but nevertheless appears strongly related to collision frequency and intensity. Likewise, the increase in perceived soreness in the hours and days post-match strongly correlate to collision characteristics across the rugby football codes. Within these findings, there are specific differences in positional groups and recovery trajectories between the codes which relate to athlete characteristics, and/or locomotor and collision characteristics. Finally, based on these findings, we offer a conceptual model of fatigue which details the multidimensional latent structure of the load to fatigue relationship contextualised to rugby. Research to date has been limited to univariate associations to explore relationships between collision characteristics and recovery, and multivariate methods are necessary and recommended to account for the latent structures of match-play external load and post-match fatigue constructs. Practitioners should be aware of the typical time windows of fatigue recovery and utilise both subjective and objective metrics to holistically quantify post-match recovery in rugby.

## Introduction

Team-based contact sports such as rugby league, rugby union, and rugby sevens are considered stochastic in nature, with players completing periods of low intensity activity (such as walking or jogging) interspersed with high-intensity actions including sprints, change of directions, rapid accelerations and decelerations, and tackles – often defined as the external load (Cunniffe et al., 2009; Coutts et al., 2010; Johnston et al., 2014a, 2018; Till et al., 2020). To adequately prepare for these demands, team sport players are required to have well developed endurance, speed and power capacities (Nédélec et al., 2012); undertaking a variety of training modalities within their programme alongside competitive matches. These varied activities impose complex psycho-physiological and biomechanical training loads onto players, leading to elevated psycho-physiological responses (defined as internal load) that result in increased post-match and post-training fatigue (Daanen et al., 2012; Johnston et al., 2014a; Vanrenterghem et al., 2017; Impellizzeri et al., 2019). Fatigue is a complex construct with a number of context-specific definitions, as well as mechanistic interpretations (i.e., central vs. peripheral) (Enoka and Duchateau, 2016; Jeffries et al., 2021). However, in the context of exposure to an exercise bout (such as rugby match-play), and in the context of this review, fatigue can be considered the disruption of homoeostasis that negatively impacts the ability to produce and apply force (Vøllestad, 1997). Fatigue processes possess acute and chronic dimensions and are therefore time-dependent, although they typically recover toward baseline following the acute effects of match-play (Aben et al., 2020). Muscle damage as a result of match-related activities has the potential to exacerbate fatigue symptoms and delay recovery (Peake et al., 2016). During the competitive season, which comprises weekly matches, prolonged fatigue, and delayed recovery is a challenge for coaches and support staff as they seek to manage, improve, or maintain athlete performance and minimise injuries. It is therefore important for players and practitioners to understand the typical (and therefore atypical) fatigue time-course following match-play, consider the measures (such as performance, physiological markers, etc.) that are currently used to quantify post-match recovery, and understand the influence of match-related actions (such as collisions) which can contribute to delays in recovery.

Athletic recovery is complex and multifaceted and involves the integration and interaction of multiple biological components such as the biochemical, hormonal, biomechanical, morphological, and psychological systems (Daanen et al., 2012; Nédélec et al., 2012). The complex interactions between these (and other) biological systems contribute to the return to homoeostasis acutely post-match due to decreased autonomic sympathetic drive, heart rate, oxygen consumption, and a return to the resting haematological and hormonal states (Daanen et al., 2012). Within skeletal muscle, other transient changes, such as the upregulation of glycogen resynthesis and an increased protein synthesis, are identified post-exercise (Hausswirth and Le Meur, 2011). In the days following exercise, recovery of skeletal muscle is determined by the psycho-physiological and biochemical stress imposed by the exercise bout (Nédélec et al., 2012), and may extend past 72 h post-exercise if the exercise bout characteristics [such as exercise duration and intensity (i.e., load)] are sufficiently high to exceed the athlete’s capacity to recovery acutely (Peake et al., 2016). To augment aspects of recovery, players may apply interventions such as hydrotherapies, cryotherapies, compression garments, and nutritional supplements during the post-match or training recovery period. These are outside the scope of this review and have been reviewed elsewhere (Howatson and Van Someren, 2008; King and Duffield, 2009; Hausswirth and Le Meur, 2011).

A fundamental factor which may influence these recovery dynamics is the induction of skeletal muscle damage as a consequence of eccentric or high-intensity exercise, known as exercise-induced muscle damage (EIMD) (Hyldahl and Hubal, 2014; Place et al., 2015; Peake et al., 2016). Rapid or repeated muscle lengthening contractions that occur during activities such as wrestling, plyometric exercise and sprinting can result in EIMD (Hyldahl and Hubal, 2014). These eccentric actions instigate myofibril disruption, which is characterised by a temporary loss of muscle force and power, increased oedema, the transient loss of range of motion, the systemic efflux of muscle bound proteins and enzymes [such as creatine kinase (CK) and myoglobin (Mb)], and delayed onset muscle soreness (DOMS) (Howatson and Van Someren, 2008; Hyldahl and Hubal, 2014; Peake et al., 2016). Following the exercise bout, activated immune and inflammatory cells (e.g., neutrophils, macrophages, and lymphocytes) migrate to the relevant sites to repair and remodel the damaged tissue, leading to a rise in pro- and anti-inflammatory markers such as the Interleukins, and C-reactive protein (CRP) (Hyldahl and Hubal, 2014; Chazaud, 2016). It has been suggested that the initial (24–48 h) loss of function (i.e., strength) that occurs following muscle damage can be ascribed primarily to intracellular calcium efflux and the associated decline in the excitation-contraction coupling pathway (Warren et al., 2002). The remainder of the strength loss that is observed thereafter can be attributed the removal of the force-generating protein structure resulting in a loss of muscle protein content (Warren et al., 2002). Repeated exposure to EIMD leads to adaptations that reduce the associated symptoms compared to prior exposures; a phenomenon known as the “repeated bout effect” (McHugh et al., 1999; Peake et al., 2016). The local and systemic effects of muscle damage and inflammation, and the strategies used to treat EIMD have been extensively reviewed elsewhere (Clarkson and Hubal, 2002; Howatson and Van Someren, 2008; Hyldahl and Hubal, 2014; Chazaud, 2016; Peake et al., 2016).

**FIGURE 1:**
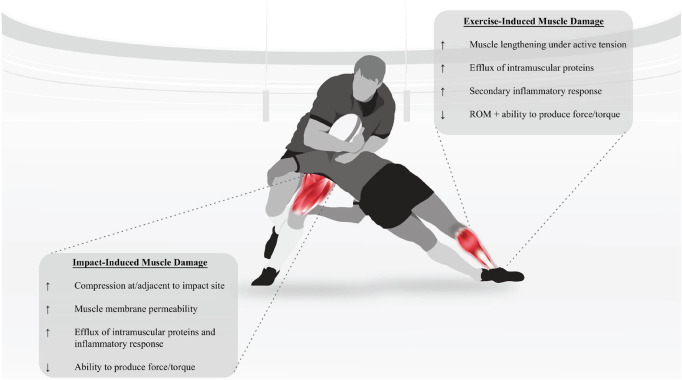
A graphical representation of a rugby collision and its relationship with exercise induced muscle damage (EIMD) (tackler) and impact-induced muscle damage (IIMD) (ball carrier), including the purported effects of EIMD (McHugh et al., 1999; Clarkson and Hubal, 2002; Peake et al., 2005, 2016; Howatson and Van Someren, 2008; Chazaud, 2016), and IIMD (Takarada, 2003; Järvinen et al., 2005; Lindsay et al., 2015b; Naughton et al., 2018). ROM, range of motion.

The collisions that characterise contact sports primarily occur during a tackle event between opposing players [i.e., ball carrier and tackler(s)] and may result in collision- or impact-induced muscle damage (IIMD) (Figure 1; Lindsay et al., 2016; Costello et al., 2018; Naughton et al., 2018). This form of muscle damage occurs via compression of skeletal muscle at and/or adjacent to the impact site (Järvinen et al., 2005). Direct muscle trauma is thought to result in tissue necrosis, systemic release of muscle-bound enzymes and proteins (such as CK and Mb), and the influx of pro-inflammatory markers (such as CRP) similar to the development of EIMD (Takarada, 2003; Naughton et al., 2018). Likewise, IIMD is believed to increase intramuscular swelling and to sensitise local nociceptors to induce the sensation of collision-related soreness (Fletcher et al., 2016; Naughton et al., 2018). These processes negatively influence performance through decreased force generation via attenuated excitation-contraction coupling (Naughton et al., 2018), and to drive an increased resting metabolic rate and a shift in tissue substrate utilisation (Costello et al., 2018; Hudson et al., 2019). It is therefore apparent that EIMD and IIMD contribute to the elevated psycho-physiological and biomechanical load imposed through training and competition in contact sports such as rugby league, rugby union, and rugby sevens (hereafter collectively referred to as rugby). Importantly, these factors have been implicated as contributing to delays in post-match recovery within rugby (McLellan et al., 2011b). There are differences in the match demands between the rugby codes. For example, rugby sevens players complete shorter duration matches over multiple-day tournaments where athletes run 1.2–1.4 km per match at an average speed of 113 m min^–1^ (Henderson et al., 2018), compared to rugby union in which athletes complete ∼5.0 km per match at velocities of 78–99 m min^–1^ (depending on position) (Tee et al., 2016). Further, rugby sevens is more homogenous for positional groups, compared to the other rugby codes (Ross et al., 2015). Whilst previous work has summarised and examined the post-match fatigue and recovery across the rugby codes, this research did not explore the influence of collision characteristics on recovery measures (Aben et al., 2020). This omission is important, given the purported contribution of collisions to elevated psycho-physiological and biomechanical load outlined above.

Rugby players typically complete repeated rapid change of direction movements, accelerations and decelerations, sprinting, and tackling during match-play (Waldron et al., 2011; Cummins et al., 2013; Oxendale et al., 2016; Till et al., 2020). Further analysis has revealed that the demands of professional match-play vary by competition (Twist et al., 2014), and differ depending on contextual factors such as technical and tactical factors, individual characteristics, and athlete positions (Oxendale et al., 2016; Dalton-Barron et al., 2020; Till et al., 2020). The actions which contribute to external load differ between positional groups. For example, in rugby league the backs positional group (i.e., the fullback, wingers, centres, half-back, and five-eight) complete a greater volume of high-speed running and high-intensity accelerations per match compared to forwards (i.e., the lock, backrowers, props, and hooker) (Cummins et al., 2013; Oxendale et al., 2016). In rugby league and rugby union, forwards complete a higher frequency of total collisions compared to backs, which has been attributed to additional defensive contacts (Twist et al., 2012; Oxendale et al., 2016; MacLeod et al., 2018; Naughton et al., 2020). The match-play differences between positional groups in rugby sevens has been described as being more homogenous (Ross et al., 2015). As described above, such elevated external loads in rugby may result in EIMD and IIMD, which has implications for recovery and may influence post-match fatigue differences between positional groups (Figure 1; Oxendale et al., 2016; Naughton et al., 2018). Similar to the recovery dynamics following EIMD being dependent on the exercise bout, temporal recovery following IIMD is related to the frequency and intensity of collisions (McLellan et al., 2011b). Therefore, IIMD could interact with EIMD and psycho-physiological and biochemical fatigue-related alterations (Vanrenterghem et al., 2017; Impellizzeri et al., 2019) to prolong post-match recovery to homoeostasis (i.e., baseline). However, whilst the relative importance of EIMD to post-exercise recovery has been examined extensively (Hyldahl and Hubal, 2014; Chazaud, 2016; Peake et al., 2016), the relevance of collisions and IIMD to post-match fatigue in rugby remains to be elucidated (Naughton et al., 2018).

Given the response-type measures used to assess IIMD and EIMD at times overlap, there is potential to confound their effects on match-related fatigue (Naughton et al., 2018), and this needs be considered when reviewing the literature on collisions. Prior work which has summarised post-match fatigue and recovery from rugby more broadly has failed to examine the relative effects of collision characteristics on recovery metrics (Aben et al., 2020). Therefore, the aim of this review is to characterise the variables that have been utilised to quantify post-match fatigue and recovery, and to specifically review the time-course of fatigue in mens rugby post-match, and the associations between fatigue and rugby match-related collisions (i.e., tackles and ball carries) for the team and in positional groups of forwards and backs. This will be reviewed in three categories; (1) neuromuscular performance measures, (2) biochemical measures, and (3) self-reported measures for mens, adult rugby players from all levels of professionalism. The time-course of post-match fatigue is considered from the acute (0–≤12 h) post-match period through to ≥72 h post-match, and the strength of the associations is considered using the recommendations of Hopkins for Pearson’s *r* product-moment correlations (Hopkins, 1997).

## Neuromuscular Performance

Neuromuscular performance refers to the ability of the neuromuscular system to produce and express force and power (Nuzzo et al., 2019). These variables have been used extensively to provide quantitative measures of fatigue across a range of areas including clinical disease models, overuse injuries, and post-exercise recovery (Nuzzo et al., 2019). Studies in rugby have quantified the post-match neuromuscular performance fatigue time-course using a variety of methods such as countermovement jumps, plyometric push-ups, and knee extension isokinetic dynamometry from the acute (<12 h) post-match period to >72 h post-match (Table 1). To further understand the purported effects of collisions on post-match fatigue, the association(s) between match-play collision characteristics (such as frequency and intensity) and the abovementioned post-match fatigue time-course and recovery measures has been investigated.

**TABLE 1:** Neuromuscular performance changes across post-match recovery.

Study	Rugby code	Measure	Group	Sample (*n*)	Δ to 0–≤12 h (%)	Δ to 12–≤24 h (%)	Δ to 24–≤36 h (%)	Δ to 36–≤48 h (%)	Δ to 48–≤72 h (%)	Δ to ≥72 h (%)
***Countermovement jump***										
Duffield et al., 2012 *^#^	RL	Height (m)	Team	11	–4.8					
Johnston et al., 2013	RL	PF (N)	Team	7	7.4	2.9	–5.4			
		PP (W)	Team		6.8	–9.4	–9.7			
Johnston et al., 2015	RL	PP (W)	Team	21	–6.5	–3.1		–1.5		
McLellan et al., 2011b	RL	PF (N)	Team	17	–18.8	–8.7		–3.3	3.4	5.0
		PP (W)	Team		–29.5	–21.5		2.5	4.6	14.0
		PRFD (N⋅s^–1^)	Team		–35.8	–27.0		1.1	18.5	17.5
McLellan and Lovell, 2012	RL	PF (N)	Team	22	–16.8	5.5		2.8	14.5	19.0
		PP (W)	Team		–31.5	–36.9		–5.6	6.7	1.8
		PRFD (N⋅s^–1^)	Team		–33.9	–21.8		2.4	7.6	9.1
Murphy et al., 2013 *^#^	RL	Height (m)	Team	9	0.0	9.7				
Oxendale et al., 2016	RL	Flight Time (s)	Team	28	–3.5		–3.6		–2.1	
Shearer et al., 2015	RU	PP (W)	Team	12	–8.0		–5.6		–2.3	
Skein et al., 2013 *^#^	RL	Height (m)	Team	11	–3.1	6.3				
Twist et al., 2012	RL	Flight Time (s)	Forwards	13		–3.3		–1.6		
		Flight Time (s)	Backs	10		–3.0		–3.0		
Webb et al., 2013 *	RL	Height (m)	Team	21	–13.8	–14.8		–9.4		
West et al., 2014a ^$^	R7	PP (W)	Team	10		–2.2				
		Height (m)	Team			–6.1				
West et al., 2014b	RU	PP (W)	Team	14	–6.9		–5.7		–2.7	
		Height (m)	Team		–9.0		–5.2		–0.8	
***Plyometric pushup***										
Johnston et al., 2013	RL	PF (N)	Team	7	2.7	–2.0	–0.6			
		PP (W)	Team		–2.6	–14.9	–21.4			
Johnston et al., 2015	RL	PP (W)	Team	21	–14.6	–10.2		–4.1		
Oxendale et al., 2016	RL	Flight time (s)	Team	28	–4.9		–7.7		–5.8	
***Isokinetic dynamometer knee extension***										
Duffield et al., 2012 *^#^	RL	MVC (N⋅m^–1^)	Team	11	–7.0					
		PT (N m^–1^)	Team		–15.4					
		RTD (Nm⋅s^–1^)	Team		–11.6					
		VA (%)	Team		–0.4					
Murphy et al., 2013 *^#^	RL	MVC (N⋅m^–1^)	Team	9	–11.0	–18.7				
		PT (N.m^–1^)	Team		–33.6	–17.5				
		RTD (Nm⋅s^–1^)	Team		–31.5	–13.4				
		VA (%)	Team		0.1	–4.5				
Skein et al., 2013 *^#^	RL	MVC (N⋅m^–1^)	Team	11	–8.1	–16.2				
		VA (%)	Team		2.0	–4.1				

All values were converted into percentage change from raw data which was extracted from tables or digitised figures or from percentage change at a given time point reported in each study. *Values are extracted from the control group condition in studies which implemented an intervention in a controlled trial. ^#^Two values were reported in the 0–≤12 h time period, with the earliest value reported post-match extracted for consistency. ^$^Only values reported with respect to day one were included as this was a multi-day rugby sevens tournament. MVC, maximal voluntary contraction; N, Newtons; Nm⋅s^–1^, Newton metres per second; N⋅m^–1^, Newtons per metre; N⋅s^–1^, Newtons per second; PF, peak force; PP, peak power; pRFD, peak rate of force development; PT, peak torque; W, Watts; R7, rugby sevens; RL, rugby league; RTD, rate of torque development; RU, rugby union; VA (%), voluntary activation percentage. Δ - indicates the change from baseline to a given time period.

### Countermovement Jump

#### Time-Course

For countermovement jump analysis, jump height and flight time have been used as measures of neuromuscular performance fatigue (Duffield et al., 2012; Murphy et al., 2013; Skein et al., 2013; Webb et al., 2013; West et al., 2014b; Oxendale et al., 2016). The magnitude of decrease in jump height between studies were variable, with five of the six studies identifying decrements in performance acutely post-match (Duffield et al., 2012; Murphy et al., 2013; Skein et al., 2013; Webb et al., 2013; West et al., 2014b) that extend to 42 h post-match (Webb et al., 2013). Conversely research in amateur players has identified jump height to recover up to 9.7% above baseline at 24 h post-match (Murphy et al., 2013; Skein et al., 2013; Table 1). The research on jump flight time appears to be less variable (Table 1). Decrements in flight time performance appearing to be largest in the 24–36 h post-match that subsequently recovers 48 h post-match (Oxendale et al., 2016).

Utilising force plates to measure indicators of post-match fatigue and recovery allows insights into how force and power variables fluctuate, and these variables display superior test-retest reliability compared to jump height and flight time (Cormack et al., 2008). Peak force decreased by up to 18.8% (McLellan et al., 2011b; McLellan and Lovell, 2012) in the acute post-match period and returned to above baseline values by 24 h in two studies (McLellan and Lovell, 2012; Johnston et al., 2013; Table 1). However, this change was somewhat variable with one study finding peak force remained attenuated to 72 h post-match (McLellan et al., 2011b). Conversely, compared to peak force, larger decrements were identified for peak power throughout the fatigue time-course, with performance decrements of up to 35.8% in the acute post-match period (McLellan et al., 2011b; McLellan and Lovell, 2012; West et al., 2014b; Johnston et al., 2015) which remained diminished to a similar extent at 24–36 h post-match (McLellan et al., 2011b; McLellan and Lovell, 2012; Johnston et al., 2013; West et al., 2014b). Typically, this returned to near baseline levels thereafter (McLellan et al., 2011b; McLellan and Lovell, 2012; West et al., 2014b; Johnston et al., 2015), although one study by Johnston et al. (2013) found a return to above baseline in the acute post-match period. Finally, peak rate of force development was attenuated to a similar extent as peak power in the acute post-match period (35.8%) (McLellan et al., 2011b; McLellan and Lovell, 2012), with performance recovering to 21.8–27.0% below baseline at 24 h post-match, and returning to baseline at 48 h post-match (McLellan et al., 2011b; McLellan and Lovell, 2012).

#### Associations

For collisions, research in rugby league has investigated the post-match fatigue time-course via the association(s) between match-related collisions dose characteristics (i.e., frequency and intensity), and countermovement jump performance changes at various times in the post-match recovery time-course. For, example, McLellan and Lovell (2012) identified large, significant inverse relationships between total collisions and collisions at higher microtechnology-derived *g* force intensity zones (Naughton et al., 2020) with changes in peak power and peak rate of force development in the acute post-match period (*r* = –0.60 to –0.73), which remained at 24 h post-match (*r* = –0.59 to –0.64) (McLellan and Lovell, 2012). Twist et al. (2012) observed similar findings across the team, and further delineated the team into forwards and backs positional groups, and by categorising collisions into offensive and defensive. Significant inverse relationships between total collision frequency and post-match performance attenuation were evident for the forwards, while no such relationship identified for backs (Twist et al., 2012). Similarly, offensive collisions were significantly related to the decrease in post-match performance for forwards, but not for backs. To the author’s knowledge, there were no studies that investigated these associations in rugby union or rugby sevens.

### Plyometric Push-Up

#### Time-Course

The generation of upper body force and power is an important factor across rugby (Gabbett and Seibold, 2013). The time-course of upper body fatigue in the post-match period has been quantified through the plyometric push-up test, analysing similar performance metrics to that of the countermovement jump; though limited studies exist and remain exclusively in rugby league (Table 1). Oxendale et al. (2016) characterised fatigue using plyometric push-up flight time and observed decrements of up to 7.7% below baseline at 36 h post-match which recovered somewhat to 5.8% below baseline at 60 h post-match.

Analysis of the plyometric push-up through force plates has been utilised in post-match recovery to investigate peak force, and peak power (Table 1). In rugby league, Johnston et al. (2013) investigated peak force and observed improvements of 2.7% in performance in the acute post-match period which subsequently declined below baseline performance by 2.0% at 24 h post-match, and then recovered to above baseline levels. However, these fluctuations are within the test-retest coefficient of variation (CV) for peak force (CV = 3.9%) (Parry et al., 2020). The largest differences in plyometric push-up performance have been examined with the peak power metric, with acute decrements up to 14.6% identified immediately post-match, which declined further to 14.9% at 24 h post-match (Johnston et al., 2013, 2015), and 21.4% below baseline at 48 h post-match (Johnston et al., 2013). Finally, a separate study by Johnston et al. (2015) identified peak power to recover to 4.1% below baseline performance at 48 h post-match.

#### Associations

There is currently one known study to investigate the association(s) between match-related collision characteristics and post-match plyometric push-up flight time performance in post-match recovery. Oxendale et al. (2016) explored this relationship at the team level in professional rugby league players and identified a moderate significant inverse relationship between total match-play collisions and plyometric push-up flight time at 12 h post-match (*r* = –0.48). The authors further explored the frequency of collisions in various *g* force intensity zones derived from player-worn 10 Hz microtechnology units (MinimaxX, Catapult Innovations, Melbourne, Australia), and identified a significant large association between moderate intensity (3–4.5 g) collisions and change in plyometric push-up performance at 12 h post-match (*r* = –0.54). All other zone correlations were statistically non-significant (*p* > 0.05).

### Isokinetic Dynamometry

#### Time-Course

The countermovement jump and plyometric push-up are considered dynamic tests for macro-level performance, whereas isokinetic dynamometry can provide insights into intra-muscular and isolated movement fatigue (Gleeson and Mercer, 1996). Isokinetic dynamometers provide varying resistance to allow constant velocity through a joints range of motion (Gleeson and Mercer, 1996). Isokinetic dynamometry has been utilised across three studies in rugby league in which knee extension performance has been assessed isometrically by maximal voluntary contraction (MVC), peak torque and rate of torque development, and electrically evoked twitch characteristics (Duffield et al., 2012; Murphy et al., 2013; Skein et al., 2013; Table 1).

Relative to pre-match performance MVC of the knee extensors was reduced by up to 11.0% in the acute ≤12 h post-match period (Duffield et al., 2012; Murphy et al., 2013; Skein et al., 2013). These differences were exacerbated when tested again at 18 h post-match as MVC was decreased by up to 18.7% (Murphy et al., 2013; Skein et al., 2013). Peak torque decreased by up to 33.6% acutely post-match (Duffield et al., 2012; Murphy et al., 2013), then partially recovered to 17.5% below baseline by 18 h post-match (Murphy et al., 2013), though this is data from only one study. Finally, rate of torque development followed a similar trajectory to other isokinetic dynamometry derived variables in being decreased by up to 31.5% acutely post-match (Duffield et al., 2012; Murphy et al., 2013), while one study reported partial recovery to 13.4% below baseline at 18 h post-match (Murphy et al., 2013).

Voluntary activation is a measure of the intrinsic contractile properties of the muscle which can be calculated by the percentage difference between the ability to activate the muscle voluntarily and the activation that is elicited by a supraphysiological electrical stimulus during rest and exercise (Shield and Zhou, 2004). Three studies analysed voluntary activation during knee extension isokinetic dynamometry following rugby league match-play (Duffield et al., 2012; Murphy et al., 2013; Skein et al., 2013) (Table 1). In the acute post-match period, changes in voluntary activation were variable and ranged from improvements up to 2.0% to decrements of up to 0.4% when compared to baseline (Duffield et al., 2012; Murphy et al., 2013; Skein et al., 2013). These values are within the test-retest CV (CV = 3.0–4.0%) and therefore may be within the minimal detectable difference (Nuzzo et al., 2019). Following this period, voluntary activation declined by up to 4.5% at 18 h post-match (Murphy et al., 2013; Skein et al., 2013). There were no studies which investigated the force production capabilities or muscle contractile properties beyond this period post-match.

#### Associations

No studies have investigated the association between post-match isokinetic dynamometry variables and the frequency or intensity of collisions during match-play.

### Summary

Across all studies investigating jump variables, the change in jump height was highly variable in post-match recovery, whilst there were more consistent findings for flight time with small decrements identified up to 60 h post-match (Table 1). Utilising jump height and flight time has inherent limitations as players may use alternate movement strategies (such as manipulating contraction time) to achieve similar performance (Cormack et al., 2008). Given such limitations, utilising sophisticated methods of kinetic profiling through force plates/platforms has been advocated (Cormack et al., 2008), and explored within rugby league post-match recovery contexts.

Through force plate analysis, the greatest decrements in countermovement jump and plyometric push-up performance were identified 24–36 h post-match, before returning to baseline within 48–72 h (Table 1). Both peak power and peak rate of force development appear to be attenuated to a greater degree than peak force, height, and flight time in post-match recovery. This observation has been noted by others (Twist et al., 2012; Johnston et al., 2013; Twist and Highton, 2013). From these findings it appears that metrics that incorporate a velocity component (such as peak power and peak rate of force development) are more sensitive to post-match fatigue than metrics that do not. Whilst the mechanism(s) of this are still not known, this has been proposed to involve preferential damage to Type II skeletal muscle fibres stimulating a shift in the force-velocity relationship to slower movement velocities which does not affect peak force generation capacity to the same extent (Johnston et al., 2013).

Isokinetic dynamometry performance decrements suggest that alterations in the properties of isolated lower-limb movements following match-play align directionally with the macro-level performance changes identified during dynamic tests, such as the countermovement jump (Murphy et al., 2013; Skein et al., 2013). However, there are financial and logistical constraints to isokinetic dynamometry as equipment is costly and bulky making it difficult to transport and setup (Gleeson and Mercer, 1996). These limitations make it impractical for practitioners to use and test large groups of players outside of research settings. Within research settings, these factors have likely contributed to the lack of studies that investigate isokinetic performance beyond the 12 h post-match period, and outside of rugby league. Given these considerations, using force plates (when available) to monitor upper-body and lower-body neuromuscular performance fatigue may be a cost and space effective alternative.

The collective findings suggest that the frequency and intensity of match-related collisions are related to the attenuation in performance post-match. Further, the observations of Twist et al. (2012) highlight a potential differential fatigue mechanism between positional groups as to the specific match-related actions that produce divergent fatigue responses (Fletcher et al., 2016). For forwards, the higher frequency and intensity of match-related collisions compared to backs could translate to a greater degree of IIMD and associated fatigue (Naughton et al., 2018). However, for backs, the decrements in lower-limb performance appear to relate to the greater frequency of high-intensity accelerations and volume of high-speed running (Oxendale et al., 2016).

## Biochemical Measures

To provide objective information about the physiological changes in response to match-play, a variety of biochemical markers have been utilised that relate to muscle damage and inflammation (Cunniffe et al., 2010; Peake et al., 2016). CK and Mb are enzymes expressed in a variety of body tissues that are involved in cellular energetics (Wallimann et al., 1992). The increased presence of the skeletal muscle-bound isoform of CK or Mb in systemic circulation is used quantitatively as a marker of muscle membrane permeability and damage (Peake et al., 2016). Following damage, a secondary inflammatory response is initiated and the systemic concentration of the pro-inflammatory CRP increases to promote breakdown and removal of cellular debris through phagocytosis (Chazaud, 2016). There are several limitations to the application of these methods which have been highlighted in the wider literature, such as the infeasibility of collecting bodily tissues with more professional athlete populations, and the large inter- and intra-individual variability that is often observed at baseline (CK inter-individual CV: 18.5–27.0%; intra-individual CV: 41.7%) and in response to sporting match-play (CK inter-individual CV: 28.1%; intra-individual CV: 30.0–34.3%) (Twist and Highton, 2013; Russell et al., 2015; Harper et al., 2016; Kellmann et al., 2018). Within rugby literature, CK and Mb are the primary markers of muscle damage that have been investigated, while CRP has been utilised as an inflammatory biomarker (Table 2). In addition, other biomarkers of neuroendocrine function such as cortisol and testosterone have been investigated with respect to their relevance to post-match fatigue and recovery (Cunniffe et al., 2010; Minett et al., 2010; McLellan et al., 2011a; Murphy et al., 2013; Lindsay et al., 2015a, b; Shearer et al., 2015). Muscle damage and inflammatory processes are known to increase oxidative stress which has been observed in rugby athletes throughout the competitive season (Finaud et al., 2006). However, there is a lack of research which has explored oxidative stress markers acutely prior to and following rugby match-play, which precluded the inclusion of oxidative stress markers in this review.

**TABLE 2:** Blood biomarkers of muscle damage and inflammation and their change across post-match recovery.

Study	Rugby code	Group	Sample (*n*)	Δ to 0–≤12 h (%)	Δ to 12–≤24 h (%)	Δ to 24–≤36 h (%)	Δ to 36–≤48 h (%)	Δ to 48–≤72 h (%)	Δ to ≥72 h (%)
***Creatine kinase* (*U/L or IU.L^–1^*)**									
Cunniffe et al., 2010	RU	Team	10	55.9	255.0		125.2		
da Silva et al., 2020	RU	Team	14	44.9	225.8		74.1	6.3	–21.0
Gill et al., 2006 *	RU	Team	23	114.5					
Johnston et al., 2013	RL	Team	7	82.5	175.5	143.8			
Johnston et al., 2015	RL	Team	21		126.0		55.0		
Jones et al., 2014	RU	Backs	13		451.5		197.1		
		Forwards	15		191.6		104.1		
McLellan et al., 2011a	RL	Team	17	50.3	211.6		96.0	83.1	46.4
Minett et al., 2010 *	RU	Team	12		133.7				
Murphy et al., 2013 *^#^	RL	Team	9	95.3	162.1				
Oxendale et al., 2016	RL	Team	28	263.3		170.2		19.6	
Suzuki et al., 2004	RU	Team	15	18.0	53.7		–14.2		
Takarada, 2003	RU	Team	15	265.5	585.2		390.5	176.6	
Twist et al., 2012	RL	Backs	10		167.9		88.9		
		Forwards	13		285.1		144.6		
Webb et al., 2013 *	RL	Team	21	36.6	167.1		91.5		
West et al., 2014a ^$^	R7	Team	10	152.8	222.2				
Skein et al., 2013 *^#^	RL	Team	11	100.0	225.0				
***Myoglobin* (μ*g/L or ng.mL^–1^*)**									
Lindsay et al., 2015a *^¤^	RU	Team	18	2589.0		–58.9			
Lindsay et al., 2015b	RU	Team	11	148.5	1.3	–24.7		–38.0	
Takarada, 2003	RU	Team	15	2091.8	191.7		33.3	8.3	
***C-reactive Protein* (*U/L or mg.L^–1^*)**									
Cunniffe et al., 2010	RU	Team	10	–2.1	121.6		201.0		
Minett et al., 2010 *	RU	Team	12		40.0				
Murphy et al., 2013 *^#^	RL	Team	9	9.8	64.1				
Skein et al., 2013 *^#^	RL	Team	11	7.3	46.0				

All values were converted into percentage change from raw data which was extracted from tables or digitised figures or from percentage change at a given time point reported in each study. *Values are extracted from the control group condition in these studies which implemented an intervention in a controlled trial. ^#^Two values were reported in the 0–≤12 h time period, with the earliest value reported post-match extracted for consistency. ^$^Only values reported with respect to day one were included as this was a multi-day rugby sevens tournament. ^¤^Raw data was provided by the author on request. IU.L, international equivalent units per litre; mg.L, milligrammes per litre; R7, rugby sevens; RL, rugby league; RU, rugby union. Δ - indicates the change from baseline to a given time period.

### Creatine Kinase

#### Time-Course

There were 16 studies that investigated the role of CK in post-match recovery (Table 2). Across all players, CK increased relative to baseline by up to 265.5% acutely post-match (Takarada, 2003; Oxendale et al., 2016), which remained elevated (585.0%) in the 12–36 h post-match (Table 2) (Takarada, 2003; Skein et al., 2013). Following this peak, there were increases of up to 176.6% relative to baseline in CK evident at 72 h post-match (Table 2) (Takarada, 2003; McLellan et al., 2011a). For positional groups, the increases in CK were larger in rugby league for forwards compared to backs at 24 h post-match (285.1 vs. 167.9%), and 48 h post-match (144.6 vs. 88.9%) (Twist et al., 2012). These differences were reversed in rugby union, as backs compared to forwards displayed the largest difference at 24 h post-match (451.5 vs. 191.6%) and 48 h post-match (197.1 vs. 104.1%) (Jones et al., 2014). These changes far exceed the typical test-retest variability highlighted above and can therefore be used to infer tissue damage.

#### Associations

At the team level, there were significant large to near perfect correlations between match-related total collision frequency and the rise in CK in the acute post-match period (*r* = 0.67), and at 24 h post-match (*r* = 0.50–0.92) (Takarada, 2003; Twist et al., 2012; Oxendale et al., 2016). In rugby league, similarly large relationships were identified at 24 h post-match when collisions were divided into offensive and defensive collision frequency (McLellan et al., 2011a; Twist et al., 2012). However, in rugby union, only offensive tackles were significantly correlated to the change in CK at 24 h post-match (Jones et al., 2014). Twist et al. (2012) explored rugby league forward and back positional groups and associations with match-related collision frequency. Significant very large associations were identified between offensive, defensive, and total collisions with the rise in CK at 24 h post-match for forwards (*r* = 0.70–0.74). Conversely, all relationships for the backs positional groups were statistically non-significant (*p* > 0.05) and had small to trivial effect sizes. In rugby union, the strongest relationships between the change in CK and collisions were seen in the acute post-match period for backs with hit-ups (*r* = 0.74), and impacts (*r* = 0.71) (Smart et al., 2008), and at 48 h post-match were for backs with tackles (*r* = 0.58), and impacts (*r* = 0.64) (Jones et al., 2014). Compared to backs, relationships for forwards between CK and these collision metrics were smaller and non-significant at these time points.

Relating microtechnology-derived collision intensity metrics to CK changes to quantify post-match fatigue and recovery has also been explored in rugby league (McLellan et al., 2011a; Oxendale et al., 2016). Oxendale et al. (2016) used a five level *g* force-derived intensity schema from light to severe, and found significant large to very large relationships between the rise of CK in the acute ≤12 h post-match recovery period and microtechnology derived collision intensities equating to zone 1 (2–3 *g*) through to zone 4 (6–8 *g*) (*r* = 0.58–0.73). McLellan et al. (2011a) utilised a six zone schema and identified significant, large to very large correlations between the increase in CK and collisions in zone 4 (7.1–8.0 *g*) to zone 6 (>10.1 *g*) immediately post-match (*r* = 0.61–0.63), and 24 h post-match (*r* = 0.63–0.77). Similarly, statistically significant large correlations between CK and collisions in zone 5 (8.1–10 *g*) and zone 6 (10.1 *g*) were identified at 48 and 72 h post-match, respectively (*r* = 0.55–0.63). There were no studies to explore these relationships in rugby union or rugby sevens.

### Myoglobin

#### Time-Course

Three studies have investigated the between-individual changes in Mb concentrations from pre-match to post match, exclusively in rugby union (Table 2; Takarada, 2003; Lindsay et al., 2015a, b). On a group level, the largest changes in Mb were identified acutely post-match with increases of up to 2589.0% identified during this period (Lindsay et al., 2015a, b, 2016). Thereafter Mb concentrations began to decline but remained elevated above baseline in one study by up to 191.7% and 33.3% at 24 h and 48 h post-match, respectively (Takarada, 2003). However, in two studies Mb concentrations declined below the pre-match baseline by 24–36 h post-match (Lindsay et al., 2015a, b).

#### Associations

Takarada (2003) is the only study to investigate the relationships between collision characteristics and Mb change post-match in rugby union. A significant, very large correlation (*r* = 0.85) was identified between tackles completed during match-play and the peak Mb concentration which occurred at 45 min post-match. There are no known studies to investigate Mb associations past this time point, or in rugby league, or rugby sevens.

### C-Reactive Protein

#### Time-Course

Two studies in rugby league and two studies in rugby union have used changes in CRP as a marker of inflammation in response to match-play (Cunniffe et al., 2010; Minett et al., 2010; Murphy et al., 2013; Skein et al., 2013). In rugby league, immediately following match-play, increases of up to 9.8% in CRP were present compared to baseline levels (Murphy et al., 2013; Skein et al., 2013), whilst a decrease in CRP was observed in one study investigating rugby union (Minett et al., 2010). Following this period across both rugby union and rugby league, CRP increased up to 64.1% and 121.6% above baseline concentrations at 16 h and 24 h post-match, respectively (Cunniffe et al., 2010; Minett et al., 2010; Murphy et al., 2013; Skein et al., 2013). In rugby union, the peak change in CRP (201.0%) occurred at 48 h post-match (Cunniffe et al., 2010; Table 2).

#### Associations

There are no studies which investigate the association(s) between CRP concentration and match-related collision characteristics such as frequency or intensity.

### Neuroendocrine Markers

Cortisol is a glucocorticoid produced by the kidney that is measured as a systematic psychophysiological stress marker (Salvador and Costa, 2009). As a stress-related hormone, cortisol acts to modulate the stress response to real and perceived stimuli, and is believed to be involved in muscle catabolism, psychological readiness prior to exercise, and aspects of threat perception, anxiety, and mood (Woolf, 1992; Salvador and Costa, 2009; Casto and Edwards, 2016). Testosterone is an endogenous steroid that is involved with anabolic processes such as protein signalling, as well as contributing to muscle glycogen synthesis (Kelly and Jones, 2013). While cortisol and testosterone are negatively correlated at times of competition, describing the purported effects between these neuroendocrine markers as inverse is imprecise as both markers have interrelated physiological effects on a variety of bodily tissues (including following exercise) outside of those described here (Casto and Edwards, 2016).

#### Time-Course and Associations

Studies have investigated both serum and salivary cortisol immediately following match-play with transient increases of up to 298.0% identified (Cunniffe et al., 2010; McLellan et al., 2010, 2011a; Minett et al., 2010; Murphy et al., 2013; Lindsay et al., 2015a, b; Shearer et al., 2015). Following this time, cortisol decreased to near baseline levels by 24 h post-match (McLellan et al., 2011a; Murphy et al., 2013) and remained at or below pre-match levels until 72 h post-match in all except one study (Cunniffe et al., 2010; McLellan et al., 2010, 2011a; Minett et al., 2010; Lindsay et al., 2015a, b). That study by Shearer et al. (2015) identified consistently increased cortisol of 30.0–52.5% immediately post-match through to 72 h post-match. Finally, McLellan et al. (2011a) investigated the associations between match-related collision frequency and microtechnology-derived (SPI-Pro, GPSports, Canberra, Australia) *g* force intensity zones with post-match cortisol and found no significant relationships at any time point.

For testosterone, increases of 13.0% relative to pre-match baseline were identified immediately post-match in rugby league which continued to rise to 67.0% above baseline at 72 h post-match (McLellan et al., 2010). During this time, these values returned to at or above 24 h pre-match concentrations, and reflected the depression of testosterone that occurred immediately prior to match-play relative to the players typical (i.e., 24 h pre-match) testosterone levels. In rugby union testosterone decreased by up to 43.9% relative to baseline in the acute post-match period (Cunniffe et al., 2010; Shearer et al., 2015) and remained attenuated by 17.9% at 24 h post-match, but recovered to near baseline concentrations thereafter (Cunniffe et al., 2010; Shearer et al., 2015). No studies have investigated changes in testosterone and the frequency or intensity of match-related collisions.

### Summary

The large increases in CK and Mb identified immediately post-match continued to remain elevated up to 36 h post-match before decreasing toward baseline. During this time, significant strong relationships between CK and collision frequency and intensity were identified. Taken together, these findings provide further indirect evidence that muscle tissue damage occurs during match-play that relates to collision characteristics (i.e., frequency and intensity) (Naughton et al., 2018). Moreover, in rugby league the forwards positional group have the highest frequency and intensity of collisions and have the higher rates of IIMD and associated post-match increases in CK, as well as decreased upper-, and lower-body performance (Naughton et al., 2020). Positional group evidence in rugby union does not support these findings, with backs exhibiting higher CK concentrations post-match, and stronger relationships with collision characteristics when compared to forwards. It is unclear why these differences exist, but they likely relate to differences in collision characteristics (e.g., contested scrums, rucks, mauls in rugby union and rugby sevens) or locomotor external loads between the rugby codes (Cummins et al., 2013; MacLeod et al., 2018). Further replication work is necessary to confirm or refute these findings.

Observations of elevated CK and Mb in the acute (<12 h) and 24 h post-match recovery periods are supported by subsequent increases in CRP, a secondary inflammatory marker which was increased when sampled at 48 h post-match. This suggests that the delayed inflammatory response following match-play follows the typical biphasic model whereby initial muscle damage and trauma signals the subsequent phase shift and escalation in inflammation (Clarkson and Hubal, 2002). In soccer, a sport without the collision component of rugby, the peak in post-match CRP occurs 24–48 h post-match before returning to baseline by 72 h (Nédélec et al., 2012; Silva et al., 2013). The largest increase in CRP were observed 48 h post-match in rugby union (Cunniffe et al., 2010), and there were no measurements at subsequent times. Moreover, Singh et al. (2011) postulate that CRP is more sensitive to muscle damage recovery involving collisions than markers such as CK and Mb. Whilst the post-match muscle damage marker time-course has been established, exploring CRP beyond 48 h post-match appears necessary to identify the peak and subsequent nadir to further characterise the time-course of post-match inflammation. This is important to establish given the repeated training and matches an athlete is exposed to, such as during the week to week in-season period.

The changes in post-match cortisol and testosterone levels, from the limited research conducted in rugby, indicate that match-play may be associated with heightened arousal, anxiety, and vigilance inducing a cortisol response (Cunniffe et al., 2010; McLellan et al., 2011a). Amongst the myriad of effects, the acute rise in cortisol post-exercise acts to signal the rise in inflammatory mediators (Peake et al., 2005), while systemic changes in testosterone act in skeletal muscle anabolism (McLellan et al., 2010; Kelly and Jones, 2013). However, the time-course of these changes suggests that cortisol increases are typically attenuated by 24 h following the stressor of match-play. Further, for both cortisol and testosterone, the return to baseline remains stable into the subsequent training week, ∼48 h post-match (McLellan et al., 2010, 2011a), a time-course which is consistent with changes across American Football (Kraemer et al., 2009). Overall, these hormonal changes suggest that training is not perceived in the same manner as match-play. A potential explanation for this difference in perception is that there are inherent differences in external load content, such as locomotion and collision characteristics, between match-play and training. Given the limited evidence linking collisions and hormonal changes, as well as the typical recovery of hormonal markers following match-play, the relevance of attempting to monitor or influence the typical fluctuations in cortisol and testosterone post-match would be limited outside of clinical conditions.

## Self-Reported Assessments

Whilst neuromuscular performance and biochemical measures have been used pre- and post-match to provide objective metrics of fatigue, other questionnaire-based methods have been used to provide subjective assessments of athlete health and well-being (Saw et al., 2016). Within team sport research, there are a number of long-form questionnaires and shorter rating scales which provide insight into a players subjective well-being and psychological readiness state (Saw et al., 2016). Rugby league studies have focussed on assessing soreness using shorter Likert-based scales with numerous scales (such as 0–6, 1–5, or 1–10) to quantify the time course of whole-body, upper-body, and lower-body soreness changes (Twist et al., 2012; Murphy et al., 2013; Skein et al., 2013; Webb et al., 2013; Fletcher et al., 2016; Oxendale et al., 2016). Further, studies in rugby league have explored associations between match-related collision characteristics and soreness at various time-points during post-match recovery (Twist et al., 2012; Fletcher et al., 2016; Oxendale et al., 2016).

### Soreness

#### Time-Course

Increases in perceived total body soreness (as assessed using single-question Likert ratings and visual analogue scales) were observed from pre- to immediately post-match when averaged within the team (Murphy et al., 2013; Skein et al., 2013; Webb et al., 2013; Oxendale et al., 2016). Within the team, soreness increases peaked between 12 and 36 h post-match (Minett et al., 2010; Murphy et al., 2013; Skein et al., 2013; Webb et al., 2013; Oxendale et al., 2016; Kupusarevic et al., 2019), before returning to baseline at 60 h post-match (Oxendale et al., 2016). One study by Twist et al. (2012) in rugby league determined positional differences in post-match whole body soreness using a 5-point Likert scale, and there were similar changes between backs and forwards at 24 h (3.5 vs. 3.2 AU) and 48 h (3.2 vs. 3.3 AU) post-match (Twist et al., 2012).

Fletcher et al. (2016) examined post-match perceived soreness longitudinally throughout a 26 match competitive season. Upper- and lower-body soreness in the backs, forwards, and adjustables positional groups were compared pre-match, and at 24, 48, 72, and 96 h post-match using a 5-point Likert scale, and there were no significant differences between upper-body and lower-body perceived soreness in the positional groups at any post-match time point.

#### Associations

Two studies in rugby league have examined the associations between collision frequency and soreness changes within the team and across positional groups (Twist et al., 2012; Fletcher et al., 2016). There were significant large correlations between match-related collision frequency and the increase in total body soreness at 24 h post-match in the forwards (*r* = 0.62), which were absent in the backs and across the whole team (*r* = 0.20–0.39) (Twist et al., 2012). These position-specific associations were mirrored when collisions were separated into offensive and defensive categories for forwards (*r* = 0.49–0.63) (Twist et al., 2012). At the same time point (i.e., 24 h post-match), there were significant correlations between total, defensive, and offensive collisions and upper-, and lower-body soreness for forwards and the team (Fletcher et al., 2016). In this research, the strongest relationships were identified for forwards and between defensive collisions and upper-body soreness (*r* = 0.42). Further significant associations between total collisions and upper- and lower-body-soreness were identified for forwards and across the team at 48 h post-match (Fletcher et al., 2016). For backs, there were no significant associations between offensive or defensive collisions and upper-body or lower-body soreness at any time point in the post-match period (Fletcher et al., 2016).

For collision intensity, a study by Oxendale et al. (2016) in rugby league explored the associations between microtechnology-derived *g* force collision intensity and soreness in the acute (<12 h) post-match period. Here, collisions in four of the five intensity zones were significantly related to total body soreness changes immediately post-match within the team (*r* ≥ 0.56). Further, the total frequency of match collisions across the team were significantly and largely related to total body soreness during the same period (*r* = 0.68) (Oxendale et al., 2016). There were no studies investigating associations with collision intensity metrics beyond this time point. The associations indicated in this section are from studies in rugby league, with there being no known studies investigating these relationships in rugby union, or rugby sevens.

### Summary

The time-course of subjective soreness changes post-match follows a similar trajectory to that of objective markers of neuromuscular performance and blood biomarkers, with the largest differences consistently observed in the 12–36 h post-match period. Similar to the objective methods (discussed above), there were strong relationships for match-related collisions and the rise in soreness in the hours and days following match-play. These associations were particularly pronounced for the forwards positional group and for upper-body soreness with defensive collisions, of which forwards complete a higher frequency per match than backs (Naughton et al., 2020). This supports the hypotheses that IIMD is a primary cause of the delays in recovery following match-play, and a potential divergent fatigue response exists for IIMD and EIMD that may be position-specific (Naughton et al., 2018).

As monitoring tools, subjective methods of data collection likely provide different but complementary information to objective markers in profiling post-match fatigue (Saw et al., 2016). Therefore, multivariate monitoring of both subjective and objective metrics is relevant when considering the players fatigue and readiness state from a holistic perspective (Colby et al., 2017). For example, an athlete may perform within their typical range (incorporating a measure of variability such as CV or through a Z-score) for a neuromuscular performance test on a given post-match recovery day, whilst their concurrent subjective soreness and wellness data may fall negatively outside their typical range (including variability), or *vice versa* (Robertson et al., 2017; Kellmann et al., 2018). Based on how an athlete scores relative to their typical response, this mismatch would then activate a notification and subsequent discussion between the athlete and supporting practitioners (Robertson et al., 2017), and additional recovery strategies or alterations in training could be implemented. Further, given the low-cost and accessible process of subjective assessment, this information can be readily collected by practitioners and particularly those who do not have access to sophisticated methods such as force platforms, isokinetic dynamometers, or blood collection. Despite their popularity, recent work has identified that a number of these single-item athlete self-report measures lack established validity and are without known measurement properties (Jeffries et al., 2020). Practitioners should therefore be mindful of the validity of the measures and scales when selecting which self-reported measurement techniques to employ.

## Conceptual Model of Fatigue

**FIGURE 2:**
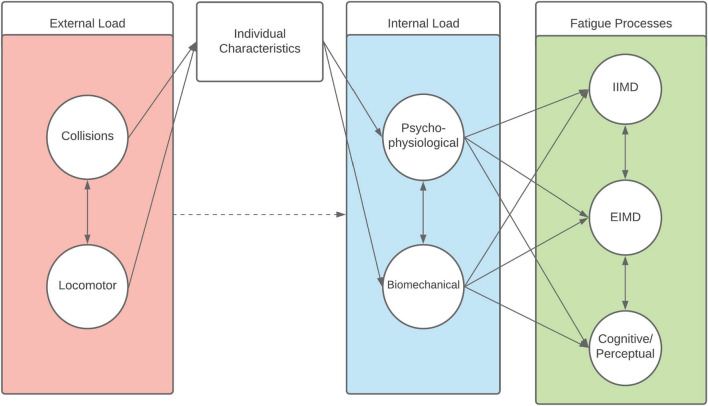
A latent structural equation model *conceptualisation* of the relationship between external load factors, individual characteristics, internal load, and fatigue generating processes [including impact-induced muscle damage (IIMD), and exercise-induced muscle damage (EIMD)] in rugby. Circles represent a construct, rectangles represent an indicator of a construct, single arrows indicate a causal relationship, double-headed arrows represent a bi-directional relationship (e.g., covariance/correlation), and the dashed arrow represents an effect which may be mediated.

This review highlights the multidimensional and complex nature of the load to fatigue paradigm in rugby. Indeed, it is apparent that post-match fatigue (as an outcome) is the consequence of the interplay between external load characteristics, internal load characteristics, and their effects on the fatigue generating processes. Based on the nature of the external loads undertaken during rugby match-play, the influence of external load on the internal load an athlete experiences, and the acute negative post-match fatigue response, these processes can be conceptualised as a model with an inherent multidimensional latent structure. Structural models are path-based methods commonly used to visualise and describe the relationships between unobserved constructs (i.e., latent variables) and observed variables within a given model. We propose that the latent structure (including both match-play content and post-match fatigue) may be appropriately described in a path diagram model from structural equation modelling (Figure 2) (Ullman and Bentler, 2003).

**FIGURE 3:**
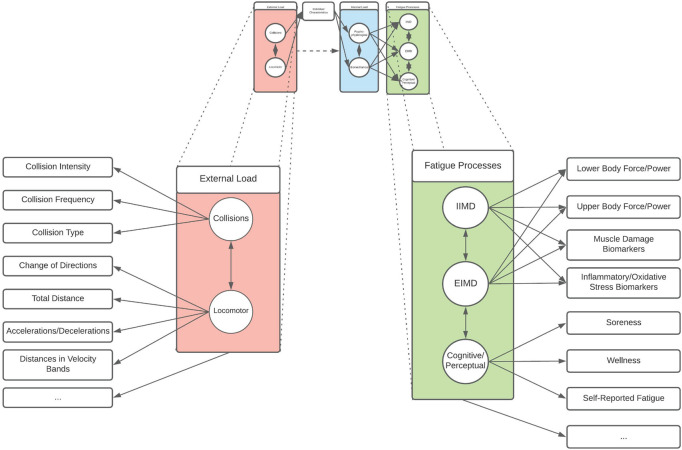
An expanded version of the conceptual model presented in Figure 2 which includes examples of functional measures of external load variables and fatigue processes such as those explored in this review. Circles represent a construct, rectangles represent an indicator of a construct, single arrows indicate an assumed causal relationship, and double-headed arrows represent a bidirectional relationship (e.g., covariance/correlation). As this model is not intended to be exhaustive, ellipses represent other functional measures which may contribute to external load and fatigue processes, but which are not included in the model.

Here, circles represent the latent constructs, square boxes represent the indicators (i.e., functional measure) of that construct, and arrows align directionally the factors with the relevant constructs (assuming a causal relationship), while double headed arrows indicate an interrelationship between constructs (Figures 2, 3). As demonstrated, collision and locomotor external loads influence the physiological and biomechanical components of internal load that is mediated through the prism of individual characteristics. Consequently, fatigue is experienced by the individual/athlete, modelled as the influence of external and internal loads on the fatigue generating processes of EIMD, IIMD, and cognitive/perceptual fatigue (Figure 2). Within each of these latent components (i.e., the circles), there are functional measures and indicators that can be used by practitioners to quantify aspects of the given component (Figure 3). This provides a multidimensional, latent structure to the proposed model. For example, collision external loads can be quantified using collision frequency and intensity metrics, whilst locomotor variables can be quantified via high-speed distance, accelerations, decelerations, and total distance metrics (Figure 3). Likewise, there are a number of functional measures which can be used to quantify aspects of fatigue (Figure 3). This model is a conceptual representation, and the list of factors and constructs, whilst being drawn from the available research, is not intended to be exhaustive. Likewise, it is not intended to be a strict mathematical interpretation of a structural equation model but rather, a conceptual framework illustrating the latent structure inherent to rugby match-play and fatigue. Whilst a variety of indicators exist, it is important that the reliability and validity of such measures is considered when selecting variables to represent an aspect of a given construct.

Given the content of this structure, the model (Figures 2, 3) is considered an application of the revised training process model of Jeffries et al. (2021). In their revised conceptual framework, Jeffries et al. (2021) describes the training effects to either be acute or chronic, and positive or negative based on the temporal nature and the overall effect on sports performance outcomes, respectively. Our model offers a specific application of this framework to rugby match-play which, given the findings of this review, should be considered a specific “acute-negative” illustrative example using the terminology described within their framework. As Jeffries et al. (2021) is a refinement of the internal/external training load models of Impellizzeri et al. (2005, 2019), it incorporates elements of this prior conceptual work alongside other models, including the seminal Bannister fitness-fatigue model (Morton et al., 1990). Further, our model aligns with that of Vanrenterghem et al. (2017) by conceptualising internal load as incorporating both physiological and biomechanical components (and subcomponents), and the work of Enoka and Duchateau (2016) by integrating perceptual and performance components of fatigue. As described, our suggested model is not intended in any way to supplant those conceptual approaches, but to offer an integrated example which has been contextualised to the specific characteristics of rugby outlined here, and elsewhere (Johnston et al., 2014a; Weaving et al., 2019b; Dalton-Barron et al., 2020; Till et al., 2020).

## Discussion

### Conclusion

Current research suggests post-match fatigue (as indicated by changes in subjective and objective measures) occurs in the days following match-play which has implications on athlete readiness into the subsequent training week. Across the performance, biochemical, and self-reported assessments, changes from the pre-match baseline are observed almost immediately in the acute post-match period (i.e., ≤12 h post-match) which peak and typically recover in the 24–72 h post-match period (see “Practical Applications” section). Larger changes in neuromuscular performance were identified following match-play in rugby league, compared to rugby union, and rugby sevens. This indicates that the post-match fatigue response magnitude is greater in rugby league, and may be specific to the different demands of match-play in each rugby code. Given that post-match tissue biomarker responses were generally similar between the codes (e.g., rugby league and rugby union), this finding may be a function of the larger research base identifying post-match changes in neuromuscular fatigue for rugby league (*n* = 10 studies), compared to rugby union (*n* = 2 studies), or rugby sevens (*n* = 1 study).

### Future Directions

Due to the added multidimensional, collision-based demands of rugby, this review focussed on the associations between these collision characteristics and fatigue type and time course which has remained, until now, uncharacterised. From this review, it is apparent that collision characteristics, such as collision frequency and intensity (Figure 3), are strongly related to the time course of fatigue measures at various time points in recovery. However, a common approach to understanding these relationships was through the use of univariate correlation analysis which includes the assumption that the individual variable is representing the complete construct [e.g., high-speed running (external load), countermovement jump force/power (fatigue)]. In Figures 2, 3, we have demonstrated a conceptual reevaluation of the external load, internal load, and fatigue processes applied to rugby as being multidimensional and multivariate. When considering match-play external loads and fatigue as multidimensional latent constructs, multivariate data analysis techniques such as structural equation modelling (or singular value decomposition derived models) (Till et al., 2016; Weaving et al., 2017, 2019a), and the mediation of effects (Mooney et al., 2011) are therefore necessary to appropriately explore and identify these relationships.

**FIGURE 4:**
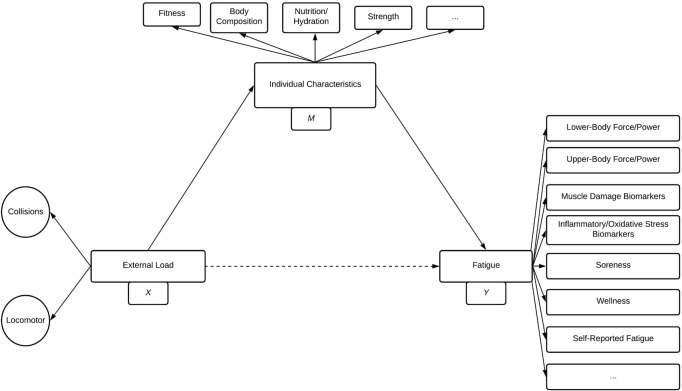
A mediation path model representing the hypothesis that individual characteristics (*M*) mediate the effect of external load variables (*X*) on fatigue variables (*Y*). This mediation model describes external load, individual characteristics, and fatigue as latent variables and includes examples of functional measures which contribute to individual characteristics and fatigue. Single-headed arrows indicate an assumed causal relationship, and dashed arrows indicate the effect may be mediated. Ellipses represent other factors which may contribute but which are not included in the model.

A considerable advantage of our conceptualisation is that it offers testable hypotheses about the effects of collision and locomotor loads in rugby. For example, according to the model (Figures 2, 3), the relative importance of fatigue types and time courses will differ depending on exposure to collision and locomotor external loads (Brustio et al., 2020; Lupo et al., 2021), and the individual characteristics of the athlete being exposed (Johnston et al., 2015). This can be conceptualised as a mediation relationship (Hayes, 2009), which is present and expanded on (Figure 4). In this mediation model, individual characteristics (such as strength, fitness, and body composition, etc.) may mediate the effects of external loads (including locomotor and collision loads) on measures of fatigue (Figure 4). Indeed, whilst prior research has shown differences in characteristics (such as strength and fitness) influence rugby post-match fatigue in junior athletes (Johnston et al., 2015), this research dichotomised the response data using a median split (i.e., stronger vs. less strong, fitter vs. less fit). This can be problematic as it is well established that dichotomising continuous data results in a loss of information, statistical power, and may result in spurious conclusions (Altman and Royston, 2006). Therefore, it remains to be determined if these (or related) factors, such as those discussed in Figure 4, are mediating this relationship. Taken together, future research should investigate the role of collisions on fatigue and recovery through both experimental models of isolated tackles and collisions (Usman et al., 2011; Burger et al., 2018), and through comparatively more sophisticated statistical approaches (such as mediation analysis and multivariate techniques) applied to observed match-play and recovery data (Mooney et al., 2011; Weaving et al., 2017, 2019a).

Given the abundance of subjective and objective methods that can be used to quantify post-match fatigue (Figure 3), practitioners need to consider which methods should be used to measure the return to homoeostasis depending on their given context. For example, methods such as force plates and monitoring of blood biomarkers of muscle damage and inflammation can be expensive and require specialised training and equipment (McLellan and Lovell, 2012; Chrismas et al., 2018; Kellmann et al., 2018). Further, tissue collection for biomarkers is invasive and may not be appropriate in certain settings. Therefore, whilst being informative as to the players’ recovery state, such methods may not be feasible in certain contexts where resources and/or athlete access is limited. In these circumstances, practitioners may consider a cost-benefit analysis and instead choose to rely on subjective assessments of recovery, and low-cost objective methods such as mobile phone applications (Balsalobre-Fernández et al., 2015; Saw et al., 2016). However, the specific methods practitioners are currently using to quantify recovery from rugby match-play in specific situations are not known, and a consensus on which methods are recommended in each of these situations is needed. Such a broad consensus on the importance of recovery monitoring methods and their feasibility to implement would inform guidelines for practitioners to select strategies that suit their environment, and availability of time and resources. Prior work in sport has utilised consensus-building methods to produce return to play guidelines following hamstring injury in soccer (Van Der Horst et al., 2017), multi-sport concussion diagnosis (Reneker et al., 2015), and a tournament preparation framework for professional and amateur golf players (Pilgrim et al., 2018). Finally, this review was undertaken on research in men’s rugby, and further research investigating women’s rugby is necessary to determine the post-match recovery timelines, and to identify if fatigue measures are related to collision frequency or intensity in the women’s game.

### Limitations

A limitation of the available research has been the inability to properly account for isometric contractions which may influence muscle damage and in turn post-match fatigue. These types of contractions typically occur during scrums, wrestling, and other static movements which are associated with a high level of exertion, and due to the lack of movement of the players during these actions, are not quantified by microtechnology devices which rely on accelerometry (Naughton et al., 2020).

Whilst collisions are associated with prolonged post-match recovery, relationships between match collisions and post-match recovery at various time points have only been explored using univariate correlational analysis (such as Pearson’s *r*) (McLellan and Lovell, 2012; Oxendale et al., 2016). Using this type of analysis in observational models is limited in that associations in such contexts cannot directly imply causation (Pearl, 2009), with causality indicated by a direct coupling of explanatory and response variables. Similarly, univariate analyses omit other factors which are expected to influence post-match recovery, including running workloads (Figure 3), playing time (Oxendale et al., 2016), and potential interactions effects between collision characteristics and external load factors (such as locomotor loads) (Johnston et al., 2014c, b; Dalton-Barron et al., 2020). For statistical analyses, the application of multiple, at times competing, univariate models increases the Type I (false positive) error rate which may produce spurious findings (Knudson and Lindsey, 2014). These factors have limited the ability to delineate the effects of collision characteristics from other confounding variables. Multivariate models and statistical mediation methods allow for the modelling of combined and interactive effects of the factors purported to influence post-match fatigue which have been outlined in this review.

Finally, during the post-match recovery time period rugby players typically apply various recovery strategies (i.e., cryotherapy, hydrotherapy, and compression garments, etc.) aimed at assisting the restorative processes toward homoeostasis (McLellan et al., 2011a; Webb et al., 2013). Whilst studies do note the application or cessation of these strategies (McLellan et al., 2011a; McLellan and Lovell, 2012), or account for these within their study design (Gill et al., 2006; Minett et al., 2010; Webb et al., 2013), this is not consistently reported, and therefore the effects of these strategies on post-match fatigue measures in the studies examined herein cannot be discounted.

### Practical Applications

Published literature indicates that rugby match-play produces decrements in neuromuscular performance, altered biochemical markers, and increased perceived soreness in the post-match recovery period. From the research it appears there are three somewhat overlapping periods or “windows of fatigue” post-match in which changes are likely to be observed for practitioners to consider. Based on these findings, the initial period extending from immediately following match-play to 12–24 h post-match in which changes in the performance, biochemical, and subjective recovery measures are likely to be identified but have yet to reach their peak, can be termed the “acute” fatigue recovery period. The subsequent period of recovery extending from 24 to 72 h post-match can be termed the “residual” fatigue recovery period, wherein changes in these measures typically peak and thereafter subsequently return to at or near baseline levels. Athletes who train during this time may exhibit higher internal loads at a given external load (Impellizzeri et al., 2019). Finally, large changes evident beyond 72 h post-match are likely to be aberrant and this period can be termed the “persistent” fatigue recovery period. Practitioners should be aware that delayed recovery may be a symptom of more ongoing fatigue that could be due to an underlying issue (e.g., overtraining) associated with a “chronic-negative” effect (Jeffries et al., 2021), and that recovery delays beyond 72 h will negatively influence athlete readiness for subsequent training and competition and should be monitored as such. However, for the majority of athletes without a confounding issue, the typical 5–7 day recovery period between matches allows sufficient time to return to their individual baselines (McLean et al., 2010).

From a collision-perspective, the delays in recovery appear to strongly relate to the frequency and intensity of collisions players engage in during match-play, and there are position-specific differences in the magnitude and specificity (i.e., upper-body vs. lower-body) of these effects. Practitioners within rugby should be cognisant that the characteristics of the collision loads that their players undertake during match-play are likely to affect recovery post-match. Practitioners should therefore ensure they are appropriately monitoring match-related collision and locomotor loads alongside their individually selected subjective and objective recovery measures.

## Author Contributions

All authors contributed to the conception, analysis, model conceptualisation, wrote, and revised the manuscript.

## Conflict of Interest

The authors declare that the research was conducted in the absence of any commercial or financial relationships that could be construed as a potential conflict of interest.

## Publisher’s Note

All claims expressed in this article are solely those of the authors and do not necessarily represent those of their affiliated organizations, or those of the publisher, the editors and the reviewers. Any product that may be evaluated in this article, or claim that may be made by its manufacturer, is not guaranteed or endorsed by the publisher.
